# Supplementary material for: Novel Inositol 1,4,5-Trisphosphate Receptor Inhibitor Antagonizes Hepatic Stellate Cell Activation: A Potential Drug to Treat Liver Fibrosis
Source: Cells. 2024 Apr 30;13(9):765. doi: 10.3390/cells13090765 (PMC11083487; doi:10.3390/cells13090765)
Supplement: Supplementary file 1 [file cells-13-00765-s001.zip › Supplementary data.pdf]

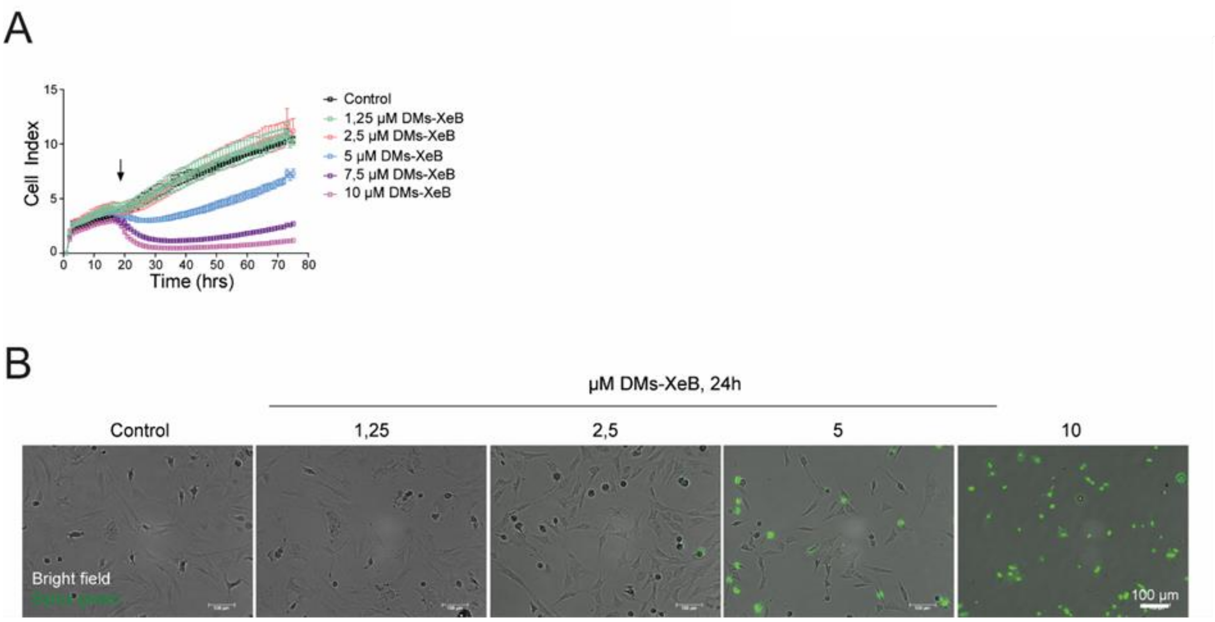

Supplementary Figure S1: Toxicity of dmXeB to primary rat HSC: (a) Proliferation rate of activated hepatic stellate cells during treatment with 0-10 μM dmXeB for 72h. Arrow represents the start of treatment. (b) Bright field (in grey) and sytox green (in green) after 72h of dmXeB treatment. n=3. Bar= 100 μm.

Tables

Table S1: List of primers and probes used. Gene, probe sequence, forward and reverse primer sequence, and species listed for each of the genes used.

Gene	*Probe Sequence*	*Forward primer*	*Reverse primer*	*Species*
acta2	CTTCACACATAGCTGGAGCAGCTTCTCGA	GCCAGTCGCCATCAGGAAC	CACACCAGAGCTGTGCTGTCTT	Rat
col1a1	TCCTGCTGGTCCCCGAGGAAACA	TGGTGAACGTGGTGTACAAGGT	CAGTATCACCCTTGGCACCAT	Rat
ACTA2	CACTCTTTCTACAATGAGCTTCGTGTTGCCC	GGGACGACATGGAAAAGATCTG	CAGGGTGGGATGCTCTTCA	Human
COL1A1	CCCCAAGGACAAGAGGCATGTCTG	GGCCCAGAAGAACTGGTACATC	CCGCCATACTCGAACTGGAA	Human
18S	CGCGCAAATTACCCACTCCCGA	CGGCTACCACATCCAAGGA	CCAATTACAGGGCCTCGAAA	Rat/human
