# Supplementary material for: Novel Inositol 1,4,5-Trisphosphate Receptor Inhibitor Antagonizes Hepatic Stellate Cell Activation: A Potential Drug to Treat Liver Fibrosis
Source: Cells. 2024 Apr 30;13(9):765. doi: 10.3390/cells13090765 (PMC11083487; doi:10.3390/cells13090765)
Supplement: Supplementary file 1 [file cells-13-00765-s001.zip › Supplementary video S1.pptx]

## Slide 1
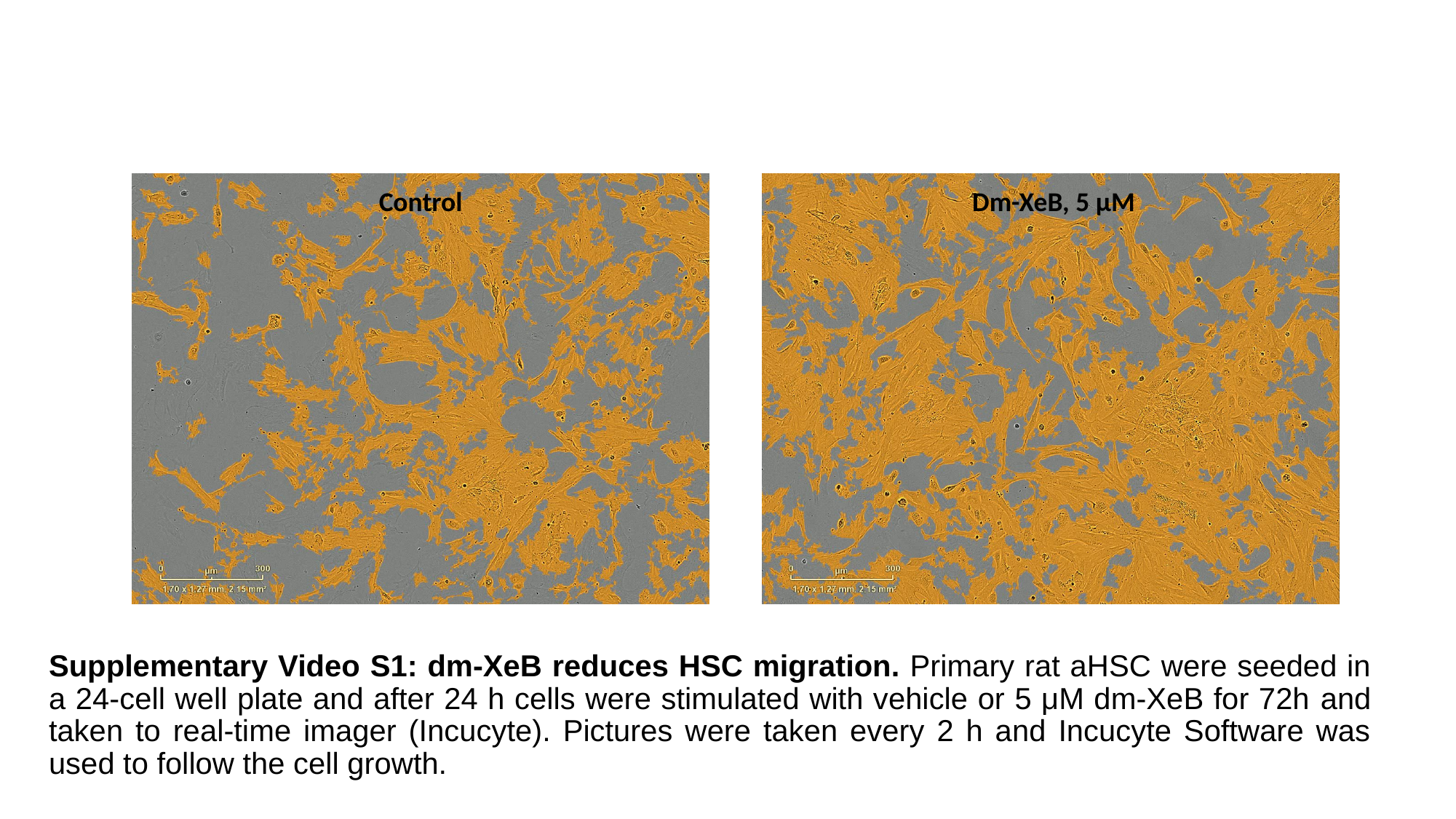

Control
Dm-XeB, 5 µM
Supplementary Video S1: dm-XeB reduces HSC migration. Primary rat aHSC were seeded in a 24-cell well plate and after 24 h cells were stimulated with vehicle or 5 μM dm-XeB for 72h and taken to real-time imager (Incucyte). Pictures were taken every 2 h and Incucyte Software was used to follow the cell growth.
